# Fibrothecoma a rare ovarian tumor: A case report

**DOI:** 10.1016/j.ijscr.2024.109771

**Published:** 2024-05-20

**Authors:** Farah Flissate, Ibtissam Bensrhir, Hounaida Mahfoud, Amina Lakhdar, Aziz Baidada, Samia Sassi

**Affiliations:** a Gynaecology-Obstetrics and Endoscopy Department, Maternity Souissi, University Hospital Center IBN SINA, University Mohammed V, Rabat, Morocco; b Anatomopathology Department, Ibn Sina University Hospital Rabat, Morocco

**Keywords:** Fibrothecoma, Ovarian mass, Magnetic resonance imaging, Surgery

## Abstract

**Introduction and importance:**

Fibroma, thecoma, and fibrothecoma collectively denote a range of non-cancerous sex cord-stromal tumors distinguished by the presence of fibroblastic stromal cells and/or cells resembling luteinized theca cells.

**Case presentation:**

In this report, we present a case study of a 52-year-old patient in whom this uncommon tumor was identified via MRI, highlighting the distinctive diagnostic and treatment considerations associated with it.

**Clinical discussion:**

Ovarian fibrothecoma tumors are infrequent, constituting less than 4 % of all ovarian tumors. Although they may manifest at any age, they are more commonly observed in elderly and post-menopausal individuals. Diagnosis hinges on clinical and paraclinical data, yet definitive confirmation is predominantly achieved through anatomopathological examination.

For younger patients, conservative surgery is usually favored, whereas peri- or post-menopausal individuals may undergo radical treatment.

**Conclusion:**

Ovarian Fibrothecoma, though rare, are typically benign tumors frequently found in older patients. Diagnosis primarily relies on histological examination. Fortunately, the prognosis for these tumors is generally favorable.

## Highlights

• Ovarian fibrothecoma is a rare benign tumor.
• It most commonly reveals by pelvic pain and abdominal distention.
• They are most prevalent among elderly and post-menopausal patients.
• Diagnosis relies on imaging techniques such as ultrasound and MRI
• The treatment is surgical and the prognosis for these tumors is generally favorable.

## 1 Introduction

Fibrothecal tumors of the ovary are uncommon neoplasms originating from the ovarian stroma, composed of differing ratios of spindle-shaped connective tissue cells and thecal cells [1].

Comprising 1 to 4.7 % of all ovarian organic tumors, they are predominantly benign. However, they frequently exhibit hormone secretion, notably estrogen, and can lead to severe complications such as adnexal torsion, necessitating prompt surgical intervention [2].

We present a case study of a 50-year-old patient where the diagnosis of a Fibrothecal tumor was suggested by MRI and later confirmed via pathological examination of the surgical specimen. Our discussion addresses the diagnostic and therapeutic strategies for these tumors, emphasizing their various clinical and paraclinical features to distinguish them from malignant ovarian tumors, where surgery is imperative for oncological reasons.

Here we report a very rare case of an ovarian fibrothecoma, highlighting the distinctive diagnostic and treatment considerations associated with it. This case report can be helpful for clinicians.

## 2 Case report

We present the case of a 50-year-old female with no prior medical history, who has had five pregnancies and five children, experiencing regular menstruation and currently in the peri-menopausal stage for two years.

She presented to our gynecological emergency department with diffuse lower abdominal and pelvic pain persisting for approximately four months. The abdominal circumference appeared symmetrically enlarged, and the pain was localized without any radiation. There were no accompanying genitourinary symptoms, and the patient reported no history of similar incidents in the past. Upon physical examination, a palpable mass was noted in the lower left abdomen.

A gynecological pelvic examination revealed a seemingly normal uterine size, a mass in the left anterior region, and no abnormal discharges or bleeding.

Initially, a pelvic ultrasound was conducted, indicating a normal uterus. However, a well-defined, hyperechoic tissue mass measuring 65 mm × 91 mm × 113 mm was observed, likely originating from the left ovary.

**Fig. 1:**
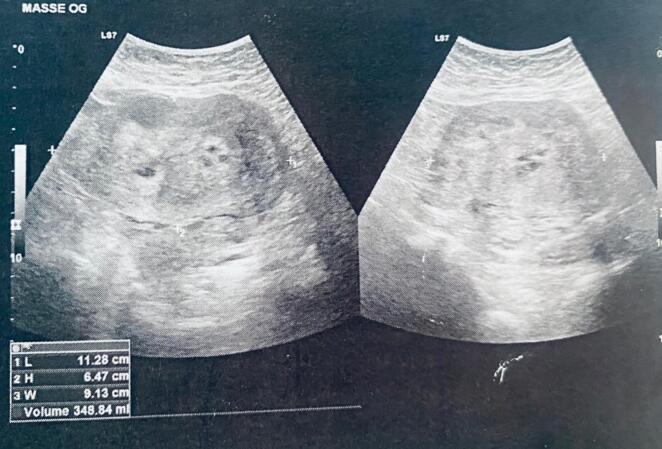
An ultrasound showing a hyperechogenic heterogeneous pre-uterine mass measuring 112 ∗ 64 ∗ 91 mm.

Doppler examination showed no vascularization within the mass, while the right ovary appeared normal (Fig. 1).

**Fig. 2:**
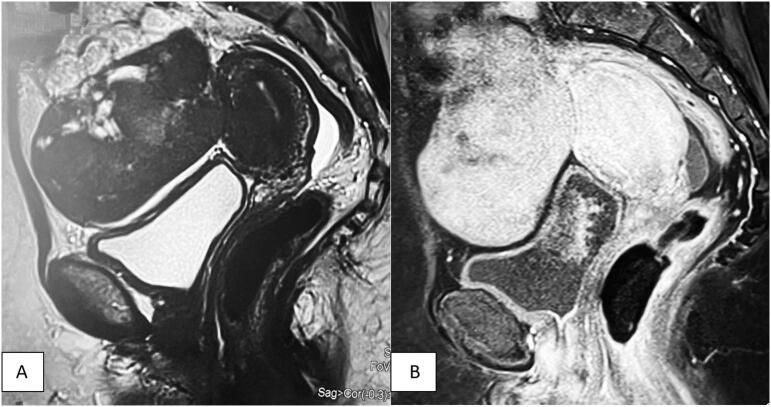
Magnetic resonance imaging of abdomen and pelvis. A: Coronal section of MRI showing the mass well defined heterogeneous in appearance with cystic areas. B: an MRI showing the enhancement after injection of the contrast.

The MRI revealed a relatively well-defined solid mass located in the left ovary, extending anteriorly and contacting the right ovary, resulting in a “Kissing” pattern. The mass appeared as a T1 hypointense signal with a pronounced T2 hypointense signal, with areas of cystic formation within. Following Gadolinium injection, the mass showed enhancement. Its dimensions were measured at 64 mm × 98 mm (Fig. 2).

Furthermore, moderate pelvic effusion was noted.

Pre-operative laboratory tests, including CA125 and HE4, were conducted and all yielded results within normal ranges. Moreover, the Roma score, utilized as a tool to evaluate the risk of ovarian cancer, was calculated at 11 %, offering reassurance regarding the probability of malignancy.

**Fig. 3:**
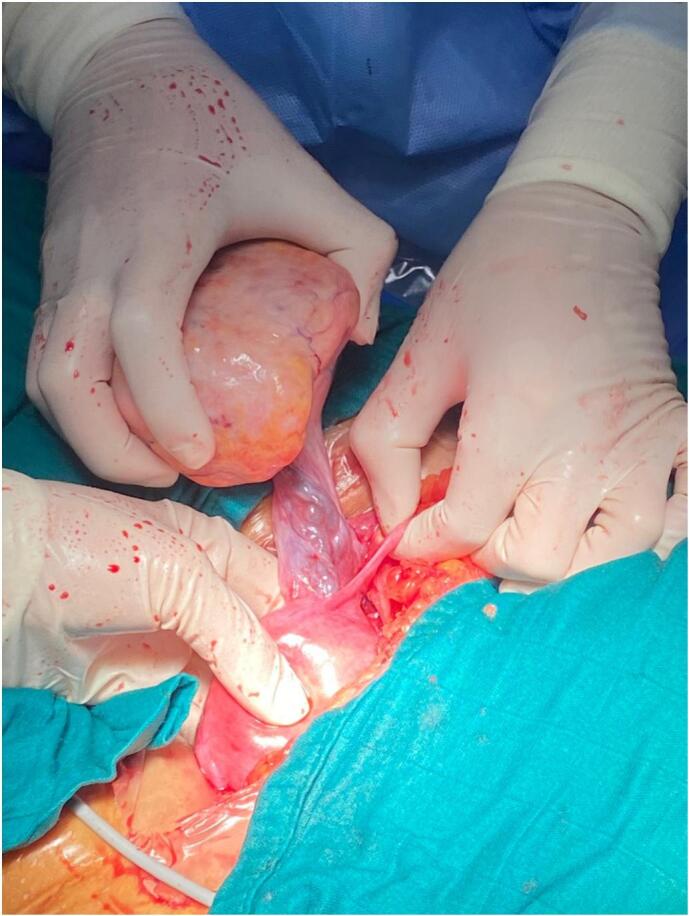
Intraoperative image prior to resection showing the left ovarian mass.

During the procedure, a laparotomy was conducted utilizing a Pfannenstiel incision. Surgical exploration unveiled a small volume of citrine-yellow ascites along with a left ovarian mass measuring approximately 100 mm × 70 mm (Fig. 3).

**Fig. 4:**
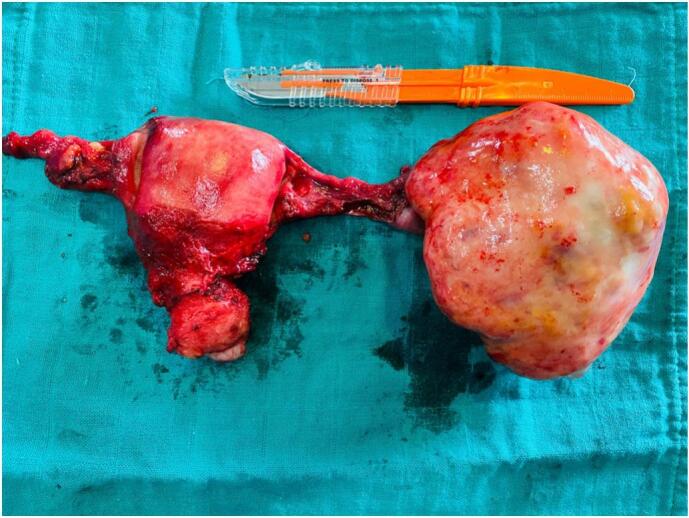
Intraoperative image showing the resected specimens (bilateral salpingo-oophorectomy) that clearly show the unilateral ovarian mass.

The uterus and the right adnexa exhibited normal appearance. Subsequently, a total hysterectomy with bilateral salpingo-oophorectomy was carried out (Fig. 4).

The patient's postoperative recovery was smooth, and she was discharged home three days after the surgery without encountering any complications.

The final pathology report revealed a mass measuring 110 mm × 105 mm × 25 mm and weighing 257.2 g, with histological findings consistent with a fibrothecoma

(Fig. 5) (Fig. 6).

**Fig. 5:**
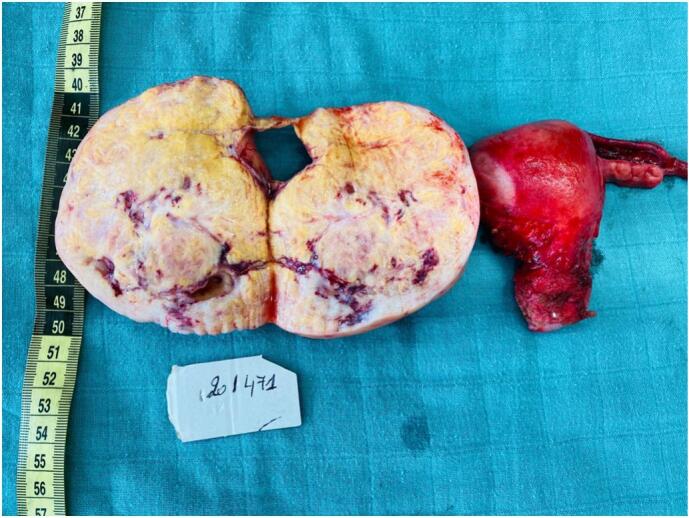
Macroscopic appearance of the tumor prior to pathological examination.

**Fig. 6:**
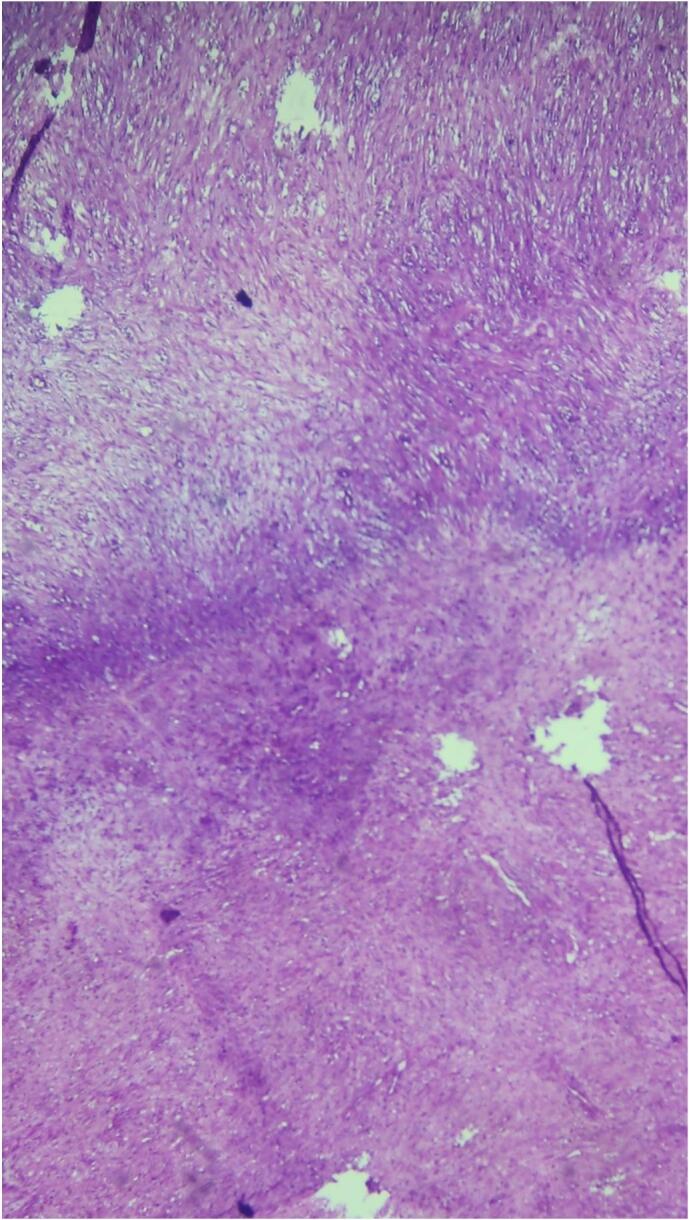
Histological section showing the appearance of a fibrothecoma.

## 3 Discussion

Fibrothecal tumors of the ovary are almost always benign; they must be distinguished from malignant thecomas and fibrosarcomas, which collectively account for less than 1 % of cases [3].

Thecomas are neoplasms originating from theca cells, whereas Fibrothecal tumors of the ovary are rare, with their frequency ranging from 1 to 4.7 % of organic ovarian tumors [2,3].

Anatomopathologically, thecomas are generally benign. Macroscopically, the tumor typically presents as a solid, yellow, and firm mass. Histologically, it is characterized by spindle, oval, or round cells forming various amounts of collagen, with a smaller proportion of theca cells [1,3].

Unilateral presentation occurs in 90 % of thecomas, while bilateral forms are often associated with Gorlin Goltz syndrome [4].

Women in their 50s, especially those undergoing perimenopause or post-menopause, have an increased susceptibility to developing fibromas, which typically exhibit non-hormonal activity. These tumors commonly present unilaterally and can be discovered under diverse circumstances. Symptoms frequently involve pelvic pain, abdominal distension, and metrorrhagia [5].

Metrorrhagia, in some cases, may be associated with an endocrine syndrome [6]. Diagnosis may occur incidentally during radiological investigations conducted for unrelated symptoms. Rarely, complications such as ovarian torsion may precipitate diagnosis.

Fibrothecoma of the ovary can also be associated with syndromes such as Meigs syndrome, characterized by ovarian fibroma, ascites, and pleural effusion, as well as rectocolic polyposis in Gardner-Richard syndrome and Peutz-Jeghers syndrome [7]. However, in our case, there is no evidence of an endocrine syndrome, digestive lesions, or Meigs syndrome.

Serum CA-125 levels typically fall within the normal range in ovarian fibrothecoma cases, although elevated levels have been reported in patients with Meigs' syndrome [8].

The diagnostic investigation of ovarian fibrothecomas primarily relies on sonography, similar to other ovarian tumors [9]. Ultrasound imaging often reveals echogenic or mixed masses, although anechoic images with posterior acoustic shadows due to fibrous tissue have also been documented [10].

On computed tomography, Fibrothecal tumors have been described as solid masses with delayed contrast medium accumulation [11].

Magnetic resonance imaging (MRI) can detect approximately 82 % of ovarian fibrothecomas, which typically exhibit a hypo signal on T2-weighted sequences due to their fibrous component. They appear as T1-iso or hypo signal masses with variable enhancement, often presenting heterogeneity. Post-contrast injection, they are typically poorly enhanced, indicating a predominant fibrous component [11].

The most common differential diagnosis is ovarian fibroma, and only anatomopathological examination can provide a definitive diagnosis.

In addition, the co-presence of ascites, hydrothorax, and high CA-125 levels lead to the misdiagnosis of ovarian fibrothecoma as another type of malignant ovarian tumor [4,7].

Treatment options may involve tumor removal alone or unilateral or bilateral salpingo-oophorectomy, with or without hysterectomy, depending on the patient's condition and the aggressiveness of the tumor [2,5,12].

In our case, a hysterectomy with bilateral salpingo-oophorectomy was performed.

## 4 Conclusion

Ovarian fibrothecomas are benign tumors, typically rare and commonly found in peri-menopausal and menopausal women. The primary symptom often includes pelvic pain accompanied by metrorrhagia. Diagnosis relies on clinical and paraclinical evaluations, particularly ultrasound, which serves as the initial diagnostic tool, occasionally supplemented by magnetic resonance imaging. However, the definitive confirmation is achieved through histological examination.

In terms of treatment, conservative approaches are preferred for young women, while older patients often undergo radical procedures. Fortunately, the prognosis for ovarian fibrothecomas is generally favorable. This study adheres to the SCARE 2023 criteria [13].

## Patient perspective

Our patient, previously experiencing persistent pain, is now able to lead a normal life, expressing satisfaction with the outcome.

## Consent

Written informed consent was obtained from the patient for the publication of this case report and accompanying images. A copy of the consent form is available for review by the Editor-in-Chief of this journal upon request.

## Ethical approval

Ethical approval is not applicable. The case report is not containing any personal information. Ethics approval is not required for case reports deemed not to constitute research at my institution.

## Funding

No funding or grant support.

## Author contribution

Farah FLISSATE, Aziz BAIDADA: performed surgery, paper writing and editing. Amina LAKHDAR: literature review, Supervision. Farah FLISSATE, Ibtissam BENSRHIR, Hounaida MAHFOUD, Samia SASSI: Manuscript editing, picture editing.

## Guarantor

Farah FLISSATE.

## Research registration number

Not applicable.

## Conflict of interest statement

The authors declare that they have no competing interests relevant to the content of this article.
